# Supplementary material for: A high-resolution mRNA expression time course of embryonic development in zebrafish
Source: eLife. 2017 Nov 16;6:e30860. doi: 10.7554/eLife.30860 (PMC5690287; doi:10.7554/eLife.30860)
Supplement: Supplementary file 6. [file elife-30860-supp6.zip › biolayout-clusters-files/Cluster216-genes.html]

Cluster216


# Cluster216: Genes

| Ensembl ID | Gene Name | Chr | Start | End | Biotype |
| --- | --- | --- | --- | --- | --- |
| ENSDARG00000007216 | abce1 | 23 | 43894304 | 43916549 | protein\_coding |
| ENSDARG00000018698 | carm1 | 3 | 34019829 | 34042706 | protein\_coding |
| ENSDARG00000015392 | dhx15 | 7 | 71039570 | 71092635 | protein\_coding |
| ENSDARG00000101578 | e2f4 | 25 | 17308796 | 17322697 | protein\_coding |
| ENSDARG00000010078 | nup133 | 1 | 53259367 | 53282163 | protein\_coding |
| ENSDARG00000042530 | nup205 | 18 | 12267318 | 12302162 | protein\_coding |
| ENSDARG00000059553 | sympk | 5 | 61199462 | 61227832 | protein\_coding |
